# A Case in Which the Vapour Bath Was Employed

**Published:** 1811-04

**Authors:** E. Kentish

**Affiliations:** Bristol


*To the Editors of the Medical and Physical Journal.*

GENTLEMEN,

THE importance of the annexed case is such that I am induced to avail myself of your politeness, in offering to communicate the effects of the Vapour Bath, which I may from time to time have it in my power to send you.

I am, Gentlemen,
Your obedient Servant,
E. KENTISH, M. D.

*Bristol, March* 3, 1811.

*A Case in which the Vapour Bath was employed.*

The treatment of the following case in a great measure occurred to me, on reading Dr. Cheyne's Treatise on Hydrocephalus Acutus, in which he has related a case of spontaneous cure taking place, in consequence of a profuse sweat breaking out over the whole head and neck; the case is as follows:—

lows:—Dr. Cheyne says—"he was informed by a friend, of a solution of this disease by sweating, and does not suppose that it is singular." His friend says, "I have frequently observed in the last moments of hydrocephalus, and when the patients were evidently moribund, a profuse sweating about the neck, or back part of the head. My Father used to mention the case of Mr. W. S.'s child, where a very profuse evacuation of this kind had the appearance of being critical. This child was attended by my Father, and an eminent Physician of Edinburgh. The disease was considered by both as hydrocephalus, and treated accordingly, till the child was thought past all hopes. The physician's visits were discontinued. A profuse sweat broke out upon the head and neck, and flowed so copiously, that the pillows had to be changed one after another, as I have heard my Father say. From that moment the child, who had lain for days in an insensible and hopeless state, is said sensibly to have recovered, and yet lives; seven years have elapsed since his recovery."—Having for some time been in the habit of immersing my patients totally in vapour, and from personal experience knowing that profuse perspiration of the head and neck might be thus excited, I determined in my own mind to put this practice to the test of experiment the first fair opportunity. Such a one occurred in the following case:—In the month of March, 1809, I was requested to visit Miss E. Y. ætat. 3. She had been feverish and ill for some days; she had great thirst, and occasional vomiting; tongue foul, skin dry, pulse very quick, eyes rather suffused, with an aversion to light, and an unwillingness to be moved; bowels moderate; she was ordered to have leeches to the temples, and take a powder with antimony and calomel every three hours. On the following day her stomach was very irritable; the medicines were frequently rejected, with a good deal of bilious matter; by adding a drop or two of tinctura opii to each powder, they were retained on the stomach, and determined to the bowels. Some of the evacuations had that dark green spinach look, termed by some, hydrocephalic stools. The powders were continued at proper intervals; the skin remained obstinately harsh and dry; therefore the bowels were acted upon to prevent congestion. Saline mixture, tamarind tea, barley water, &c. &c. were given as occasion required. The fever having gone on in this train for near a fortnight, she was in the evening suddenly attacked with convulsions; the eyes were distorted, the limbs quivering, and the whole appearance such as to threaten instant dissolution. The head was shaved, leeches applied, and afterwards a large blister: an aperient powder with jalap and calomel calomel was also administered. The united effect of these means appeared for the time to check further effusion; the head was dressed with savine ointment to keep up the discharge. Having proceeded thus for about a week, there appeared no convalescence, the eyes were still distorted, and she was becoming comatose; I was, therefore, apprehensive of further effusion in the head, which would have undoubtedly terminated the existence of my patient; the sutures of the head had never been closed, and they now appeared more open; under these circumstances I communicated my fears to the parents, at the same time mentioning the case in Doctor Cheyne's treatise, saying, I wished to try the effect of exciting, by art, the same discharge, which nature had pointed out in curing that patient. They readily came into my views, to fulfil which, she was folded in a wrapping gown, and in the arms of her nurse, conveyed in a carriage to the bath house, which was at some distance; she was put into the vapour bath in the arms of her servant, and remained totally immersed in a vapour (impregnated with rosemary) at 102 deg. for twenty minutes; during the time she was in the bath, she was quiet, and appeared inclined to sleep; though her body was well moistened by the condensed vapour, yet it did not appear that she had perspired much; the whole surface of the body was red, and the superficial vessels turgid; she returned home in the same manner she came. During the night she had some perspiration in the palms of the hands, and the skin, in general, had a more soft and pleasant feel, she passed a better night, and appeared evidently relieved; this encouraged us to proceed; the bath was repeated the following day, when it was carried up to 105 deg. and continued longer; this time she evidently perspired in the bath, after which she passed a good night; her head was much relieved; on the following morning she appeared more lively, and played a little with a doll, which was what she had not done before during her illness. From the first application of the bath she was evidently benefited, and from the second I date her perfect convalescence; she continued to use the bath every third day for some time; she also took some tonics, these with attention to her bowels, and afterwards the alternate use of the cold shower bath, with the vapour bath, restored her to perfect health.

### OBSERVATIONS.

Although in the commencement of this case it had not all the characteristic symptoms of hydrocephalus, yet, as the disease advanced, there was every reason to suppose effusion had had taken place on the brain; the convulsions, the distortion of the eyes, and the unclosed head, with the coma, all induced me to form this opinion; the treatment of the case had been pretty nearly the same as it would have been, had I in the first instance regarded it as hydrocephalus; plentiful bleeding, and blistering the head, with continued doses of calomel and antimony, which very sufficiently acted upon the bowels. Unavailing as these means were to excite absorption, I should not have been induced to have added a warm bath; fearful that the increased circulation would have determined more blood to the head; but the case already quoted from Dr. Cheyne, and knowing that sweating of the head and neck could be excited by a vapour bath more certainly, than by any other known means, induced me to administer it in this case, the result of which I am relating. Doctor C. W. Quin mentions, in his treatise on the dropsy of the brain, two cases in which nature relieved herself by great and continued discharges of perspiration from the head; one continued for some years. Most of my medical readers are aware of the inefficiency in severe cases of the usual means, viz. bleeding, purging, blistering, mercury, diuretics, and cold applications to the head. I have, therefore, much pleasure in performing the duty of recommending to their notice the effects of vapour when applied to the head, in such a manner as to produce sweating, in cases threatening effusion.

